# Selections from My Case-Book

**Published:** 1873-06

**Authors:** J. S. Todd

**Affiliations:** West Point, Georgia


### SELECTIONS FROM MY CASE-BOOK,

*Illustrating the Efficacy of Iodide of Potassium in Asthma, and the Beneficial Effects of the Hypodermic Injection of Morphta Directly over the Seat of Pain.*

BY J. S. TODD, M.D., WEST POINT, GEORGIA.

How iodide of potassium acts in relieving asthma, I will leave for others to decide. Too much space is consumed in nearly every "scientific article" in vague conjectures. It is a fact no less true than lamentable, that while we are better surgeons, histologists and *diagnosticians*, than our brethren of thirty years ago, we excel them very little in therapeutics. Indeed, we seem to have in some manner lost sight of remedies; our zeal for curing has been swallowed up in determining the disease and its pathology.

Who has not read a long article—on fibrinous deposits, and its mode of detection on the valves, for instance—and having finished, much edified no doubt, but still constrained to ask himself the inevitable question, *cui bono?* I assuredly say with Coke, "Blessed is he who knoweth the reason of things;" but because I can not tell why mercury cures syphilis, quinine ague, or that the scabies insect is so certainly destroyed by sulphur, that is no reason they should be eschewed.

Begging pardon for this digression, let me introduce Mrs. J., aged fifty-four years; housewife; white. She has been for the last seventeen years a sufferer from asthma. It comes generally in April, and lasts her never less than seven weeks, and frequently sixteen. During this period she is never at any time entirely free from it. Last year she was unable several times to lie down for ten days and nights together. She has tried all the usual, and a great many unusual remedies, with varied results; nothing has done her good more than three times—that is the same agent would lose its effect after being used twice—and all were of a temporary nature. I treated her last year. She was confined to her bed three months.

April 7, 1873.—She was taken very violently; tongue furred, and some fever; gave her calomel (which acted finely) and a Dover; she did not lie down during the last twenty-four hours.

April 8.—Gave potass. iodide, ʒ iss ; tinct. lobelia, ʒ ij ; aqua, ℥ i. Teaspoonful every four hours, which contained to each portion ten grains of potass. iodide.

April 9.—Looks and feels better, but still can not lie down ; continued the above.

April 10.—Slept in a recumbent posture very well ; no wheezing ; gave her a prescription containing five grains of iodide of potassium and the fourth of a drachm tinct. lobelia, thrice daily.

April 25.—The lobelia caused her appetite to fail, and considerable nausea all the while, so I made another prescription in which it is left out, and for it and the water, tinct. quassia is substituted, as a vehicle and tonic, with the iodide of potassium.

April 27.—Had a regular malarial chill, (*i.e.* one followed by fever and sweat); during the congestive stage she suffered terribly with asthma, but it wore off as the third or sweating stage came on ; gave quinine, but continued last prescription.

May 4.—Seven days later had another chill, and the same trouble experienced during the congestive stage as during the last ; gave quinine again, and shall guard against another.

May 17.—Went off to-day on a visit ; has taken no medicine, save a little bromide of potassium occasionally at night for restlessness, since May 12th. She has had occasional attacks of shortness of breath, but no symptoms sufficient to keep her from lying down or confined to her room.

May 20.—Saw her to-day; well ; appetite good, etc.

Mrs. G., aged forty years ; housewife ; white. Has had occasional attacks of asthma for the past five or six years, generally two or three months apart. Eight months ago, after recovering from an attack, I put her on iodide of potassium, which she continued for a month, and now when she feels any premonitions of an attack she takes it in ten grain doses every four hours. She has had no spell of it since the treatment was begun.

I deem it but justice to Dr. J. S. Weatherly, of Montgomery, Alabama, to say that to him am I indebted for the suggestion which led to the treatment detailed.

The following extract from *Headland on the Action of Medicines*, led me to infer that pain might be relieved more speedily by injecting the anodyne over the seat of pain, for between all the deep-seated organs and skin there is almost direct nervous and in some places vascular connection. I also thought that we would run less risk of narcotism; in fact relieve pain, and not necessarily produce sleep, as is almost necessarily induced by the large quantities of morphine required in those most painful of all affections, nephritis and bilious cholic. "We know that the nerves are very much under the influence of mechanical impressions, upon which depend the phenomena of two at least of the five senses—those of hearing and touch—as we should find also the other three if we understood them better. We know also, if we accept the atomic theory, by which so many chemical phenomena are cleared up and explained, we must admit a certain definite and peculiar arrangement and shape to the ultimate particles of every compound body. Such considerations render it possible that the ultimate particles of a stimulant medicine may be of such a nature as to irritate, or to refuse to coincide with the ultimate molecules of the sensitive nerve with which they come in contact; and those of a sedative may, on the other hand, be so shaped and arranged as to dovetail with those particles, and by extinguishing, as it were, their salient points, to cloak their vital sensibility."

The whole nervous system is not affected when we have a tic douloureux, a toothache, or sciatica. If we can "cloak the sensibility" of these particular sentient fibres, is it not best to do so? Why deaden the entire nervous sensibility, when only a part is affected? Shall we deluge a town to put out a fire that is raging on but one square?

Maj. M. E. E., aged fifty-one years; white; merchant. Suffering from kidney cholic. Gave morphine, per orem—rejected in half an hour. I was called to see him at 3 A.M. February 13, 1873. Injected one-fourth grain in arm, sensibility somewhat deadened but still, as he expressed it, "killing;" from that time to 9 A.M. I injected, at intervals of an hour, one grain. I then introduced same quantity (one-fourth grain) *over the seat of pain*, and he was entirely relieved in less than a minute. The previous injections, all in the arm, had only very slightly palliated his anguish. Six hours after, regularly for thirty-six hours, did the pain return with all its fury, and was, in every instance, promptly subdued by an injection *over the seat af pain.* He recovered without any untoward symptom.

C. T., colored; carpenter; aged fifty-eight years. Suffering more severely than I ever saw any human being, with nephritic colic. Morphia in large doses was given by mouth, which was rejected; injected in arm with no benefit, but almost immediately after injection over *seat* of pain, was relieved. The stone in his case occupied twelve hours in its passage, and it was necessary to repeat the injection twice, at four hours interval, over seat of pain; after each injection he complained of no pain.

W. C. C., aged twenty-seven years; merchant. Subject to attacks of bilious cholic. In this case I could distinctly feel the stone. He was, of course, racked with pain. I immediately injected morphia over the seat of pain, with the happiest results. In this case had to do so but twice.

I could go on multiplying these cases, but deem it unnecessary. I have lately intentionally injected into the arm and over the seat of pain, alternately, and watched the effect. In every case at least double the amount was requisite when a distal point was selected; sleep was induced, and nausea and vomiting more frequent and distressing after the opiate began to "die out."

I would observe, in this connection, that medicines when used hypodermically should never be injected, if we wish them to act quickly, into the cellular tissue. Inject into the rete mucosum; split as it were the skin, do not penetrate through it.

